# Prognostic Factors among Brain Metastases in Newly Diagnosed Ovary Cancer: A Large Real-world Study

**DOI:** 10.7150/jca.44494

**Published:** 2020-05-18

**Authors:** Sujuan Xi, Zaiyi Li, Quan Guo, Wenjing Lin, Xiaokun Liang, Lin Ma

**Affiliations:** 1 The Reproductive Medical Center, The Seventh Affiliated Hospital of Sun Yat-sen University, Shenzhen 518107, China; 2 Department of gynaechology, Shengjing Hospital Medical University, Shenyang, China; 3 Shenzhen Colleges of Advanced Technology, University of Chinese Academy of Sciences, Shenzhen, Guangdong, 518055 China

**Keywords:** Ovarian cancer, Brain metastases, Prognosis, associated factor, SEER

## Abstract

**Background**: Population-based data on the prognosis of brain metastases at initial diagnosis of ovary cancer (OCBM) are currently lacking. Besides, the effective treatment for OCBM patients is still controversial now. The study aimed to explore the prognostic factors among OCBM.

**Methods**: We retrospectively reviewed the OCBM patients from the Surveillance, Epidemiology, and End Result (SEER) database of the National Cancer Institute to investigate predictors of the presence of OCBM and its' prognostic factors related to all-cause mortality. We employed multivariable logistic and Cox regression analysis. Furthermore, to minimize the impact of potential confounding factors, we conducted a 1:1 propensity score matching (PSM) analysis.

**Results**: A total of 29,512 cases of OC patients entered into the study, including 89 patients with brain metastases of ovarian cancer, which accounted for 0.30% of the entire cohort and 12.02% of the metastatic disease subset. We identified eight factors, including laterality, histology, surgery, radiotherapy, chemotherapy, and extracranial metastatic sites to bone, liver, and lung, as predictors of OCBM based on multivariable logistic regression among the entire cohort. The median survival time of OCBM was 2.0 months, and the interquartile range was 2.0-10.0 mo. The patients who received comprehensive treatment had better prognosis. Based on the multivariable Cox model, marital status, surgery, chemotherapy, and extensive therapy (including RSC, SC, and RC) were identified as predictors of OS. Besides, a new factor (brain metastasis) was identified by 1:1 PSM -based multiple Cox regression, apart from the above prognostic factors for OS.

**Conclusions**: This study provided a population-based estimate of the proportion and prognosis for newly diagnosed ovary cancer with brain metastases. These findings may add materials to guidelines for preliminary screening and optimal treatment of OCBM patients.

## Introduction

Ovarian cancer (OC) accounts for about 2.5% of total malignancies among women, however, causing nearly 5% of all cancer-related deaths due to its high mortality rate 1-3. In the year of 2018, there are approximately 22,240 newly diagnosed ovary cancer cases, and about 14,070 estimated deaths occurred 2. OC patients were often found at an advanced stage (III or IV) when diagnosed, taking up over 60% of the total number, with synchronous distant metastases 4. That might partially explain their higher mortality 5. According to the latest study, the brain ranked the fifth common metastatic site behind the liver, distant lymph nodes, lung, and bone 6. Brain metastases from ovary cancer always portend a grave prognosis, leaving only a few months of survival after diagnosis 7. The incidence of ovarian carcinoma with brain metastases (OCBM) was reported to range from 0.29% to 12.0 %^8^-^13^.

However, clinical data on ovarian cancer brain lesions are scarce. The scarcity and low prevalence of OCBM make it challenging to draw firm conclusions or reach a consensus on the optimal therapy. Moreover, the brain remains a “sanctuary site” for the complex blood-brain barrier structure, limiting the penetration of drugs and causing problems for treatment. Treatment strategies are still controversial, and their efficiency needs further evaluation. Besides, there are still many things unknown about the predictors as well as prognostic factors of OCBM. There is a lack of reliable and multicentral population-based series study of the incidence of brain metastases in ovary cancer diagnosis 14. Based on the SEER database, this article focused on the risk and prognostic factors of de novo brain metastases in ovarian carcinoma.

## Materials and Methods

### Database

The SEER database has recorded cancer data of 18 population-based cancer registries, covering nearly 30% of the U.S. population 15, 16. It has collected information on patient clinical demographics, primary tumor site, tumor morphology and stage at diagnosis, the first course of therapy, and follow-up for vital status (https://seer.cancer.gov/about/overview.html). We employed the SEERStat software to determine eligible patients for analysis, and extracted the information on cancer incidence from the official website (https://seer.cancer.gov/). Since the SEER database began to record metastatic information including brain metastases from the year of 2010. We collected the information about OCBM from 1 January 2010 to 31 December 2016.

### Study population

In the SEER database, a total of 40,860 patients were diagnosed with ovarian cancer, excluding 395 less than 18 years old. Among them, we identified 38,021 patients with clear brain metastasis information. We excluded those patients with other cancers, or unknown sequence numbers, or diagnosed by autopsy or via death from the analysis, leaving 29,512 active follow-up patients in the final cohort for further analysis. Of these, 3546 patients diagnosed with metastatic disease to any distant site, and 89 patients diagnosed with OCBM.

**Figure 1:**
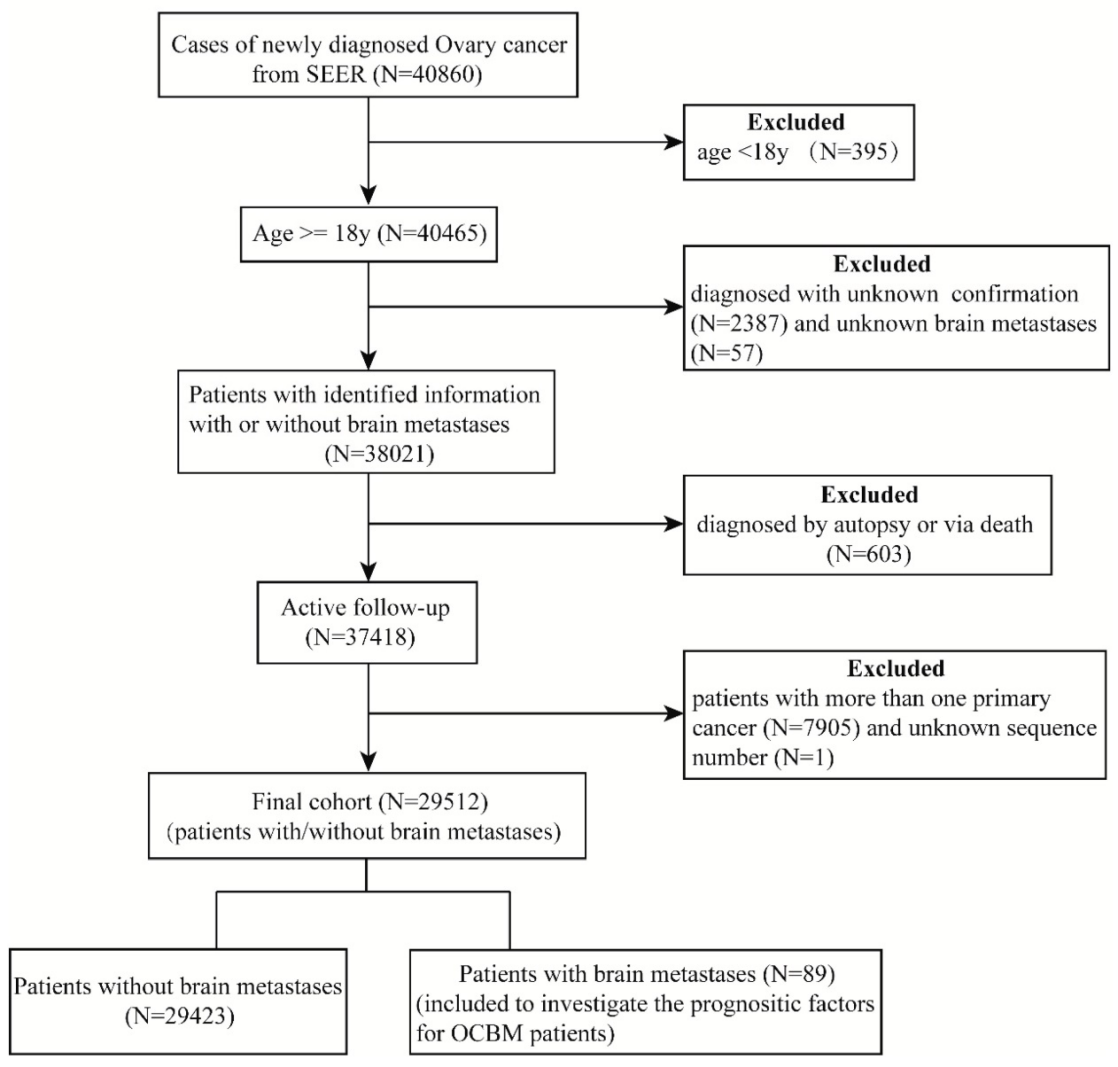
Data extraction flow chart from the SEER database.

In the entire cohort, the percentage of metastatic disease was 12.02%, and the brain metastasis was 0.30%. The data extraction flowchart was shown in Figure 1. The inclusion criteria were as follows: age elder than 18 years old; ovary cancer as the only primary malignant tumor; with clear information on brain metastases; with active follow-up. Exclusion criteria included the following requirements: age younger than 18 years old at diagnosis; with cancer other than ovary cancer; no clear information about brain metastases; diagnosis based on the death certificate or autopsy; no definite survival time; no active follow-up.

### Statistical analysis

The baseline characteristics of the population were shown in Table 1. We stratified the patients by age (18-58, 59+ years old), race (white, black, others and unknown), origin recode (Spanish-Hispanic-Latino and Non-Spanish-Hispanic-Latino), laterality (unilateral including left, right, only one side unspecified, bilateral and unknown), sequence of radiotherapy and surgery (radiotherapy before surgery, radiotherapy after surgery, radiotherapy before and after surgery and others), treatment and other sociodemographic information, such as marital status (married, single, divorced, widowed and unknown), insurance situation (yes, no and unknown), residence type (rural, urban, metropolitan and unknown). Based upon the 7th edition of the American Joint Committee on Cancer (AJCC) Cancer Staging Manual, we classified T staging (T1, T2, T3 and unknown) and N staging (N0, N1 and unknown). Tumor staging was classified into 5 categories: I, II, III, IV and unknown, according to the 7^th^ edition of the AJCC Cancer Staging Manual and SEER combined stage group as well. Surgery was classified as unilateral/bilateral (salpingo-) oophorectomy (25-28, 35-37, 50-52, 55-57, 80); debulking/cytoreductive surgery (60-63), others (17, 70-74, 90) and no surgery (0, 99). We reclassified treatment into 8 categories: patients receiving all three therapeutic approaches including chemotherapy, surgery and radiotherapy(CSR), radiotherapy and surgery (RS), chemotherapy and surgery (SC), radiotherapy and chemotherapy (RC), only receiving radiotherapy (R), only receiving surgery (S), only receiving chemotherapy(C) and patients received none of any treatment above (Others) as other investigators^16^. We defined the resident type by the county attributes from the 2003 US Department of Agriculture rural-urban continuum codes.

We stratified baseline clinical data (Table 1) and calculated the incidence proportion of the OCBM patients among the metastatic disease cohort and the entire cohort as well. Besides, we employed univariable and multivariable logistic regression analysis to identify potential predictors for the incidence of brain metastases at the diagnosis of OC. Survival estimates were based on the Kaplan-Meier method. Univariate and multivariate Cox regression analysis were employed to identify potential covariates associated with increased all-cause mortality. In the Cox regression model, we constructed 2 models for analysis. The first model included the following variables: marital status, histology, surgery, radiotherapy, chemotherapy, and insurance type. In the second model, we used treatment (CSR, RS, SC, RC, R, S, C, Others) to replace three therapeutic variables (surgery, radiotherapy, chemotherapy), and keep other variables the same with the model 1, as other investigators^16^.

Additionally, to further control the potential confounding factors across groups, we employed a 1:1 propensity-score matching (PSM) analysis based on whether brain met or not to re-examine the impact of brain metastasis among ovarian cancer on the overall survival. In this study, a 1:1 pair matching was performed by the nearest neighbor method to generate a matched pair among the brain met group and without brain met group. A chi-square test was employed to compare categorical variables across groups.

The statistical analyses were performed using SPSS statistical software (version 22.0) and RStutio (version 1.1.453). A two-sided *p*-value < 0.05 was considered statistically significant.

## Results

### Patient characteristics and Proportion

A total of 29,512 patients diagnosed with ovary cancer entered into our study, including 89 patients diagnosed with OCBM, and the median age was 62. The clinical baseline characteristics were demonstrated in Table 1. Among 29,512 patients diagnosed with ovary cancer between 2010 and 2016 in the U.S., 3546 (12.02%) presented with metastatic disease, and 89 (0.30%) patients presented with synchronous brain metastases when diagnosed.

According to the univariable logistic regression in the entire cohort Table S1, fourteen factors achieved significance (p <0.05), including age, laterality, histology, T, N, surgery, radiotherapy, chemotherapy, radiation sequence with surgery, the presence of bone, lung and liver metastasis, CA125, and insurance type. As is shown in Table 1, the elder age (0.36%) was prone to presenting brain metastases than younger age (0.23%) (P<0.05), the unknown laterality (0.72%) was more likely to be OCBM than unilateral laterality (0.42%, P<0.0001), the tumor staging grade IV (1.06%), and unspecified (0.09%) got more risk of OCBM compared to grade III (0.01%, P<0.0001, respectively), unknown N staging (0.75%) was more likely to develop OCBM than N0 (0.22%, P<0.0001) among the entire cohort. For histology, unspecified (2.02% and 8.37%) had a higher proportion of brain metastasis than epithelial neoplasms (0.25% and 2.21%) (P<0.0001). In the treatment, the absence of chemotherapy (0.53% and 4.27%) had a significantly higher likelihood of developing brain metastasis than chemotherapy treatment (0.20% and 1.69%) (P<0.0001). Besides, the brain metastases rate of uninsured patients (0.64%) was higher than insured patients (0.28%) (P<0.001) among the entire cohort, which might due to the patients with insurance might suffer less risk to develop metastatic diseases for they could receive more medical intervention.

We then put these factors into multivariable logistic regression and found that laterality, histology, surgery, radiotherapy, chemotherapy, bone met, liver met and lung met had significance among the entire cohort and histology, radiotherapy, chemotherapy, bone met, liver met and lung met achieved significance among the subset with metastatic disease cohort. According to the multivariable logistic regression based on the entire cohort (Table 2), laterality (bilateral vs unilateral, OR, 2.251; 95%CI, 1.206-4.204; P=0.011), unspecified histology (vs epithelia neoplasms; OR, 2.568; 95%CI, 1.246-5.293; P=0.011); surgery no surgery (vs U/BSO; OR, 4.047, 95%CI, 1.702-9.624; P=0.002); radiotherapy (vs no radiotherapy; OR, 32.268; 95%CI, 15.423-67.508; P<0.0001) and chemotherapy (vs no chemotherapy; OR, 0.503; 95%CI, 0.287-0.881; P=0.016), bone met (vs no bone met; OR, 5.095; 95%CI, 2.737-9.488; P<0.0001) and unknown (vs no bone met; OR, 7.271; 95%CI, 1.811-29.190; P=0.005), liver met (vs no liver met; OR, 1.072; 95%CI, 0.581-1.977; P>0.05) and unknown (vs no liver met; OR, 5.266; 95%CI, 1.706-16.250; P=0.004) , lung met (vs no lung met; OR, 5.328; 95%CI, 3.124-9.085; P<0.0001) and unknown (vs no lung met; OR, 1.627; 95%CI, 0.369-7.176; P=0.521). The multivariable logistic regression results of the subset with metastatic disease were also presented in Table 2.

### Survival

The univariate analysis of all-cause mortality among the entire cohort as well as the subset with brain metastases were presented in Table S2. Seven factors were significantly associated with overall survival among brain metastases subset (p <0.05). They were marital status, histology, treatment, surgery, radiotherapy, chemotherapy, and insurance situation. On Cox regression analysis among brain metastases in the model 1, the results (Table 3) showed that divorced (vs married; HR, 2.688, 95%CI, 1.102-6.560; P=0.030), no surgery (vs U/BSO; HR, 3.712, 95%CI, 1.519-9.075; P=0.004) were significantly associated with increased all-cause mortality and chemotherapy (vs no chemotherapy; HR, 0.341, 95%CI, 0.169-0.687; P=0.003) reduced the risk of death. While single marital status (vs married; HR, 0.679, 95%CI, 0.351-1.313; P=0.249) and cytoreductive surgery (vs U/BSO; HR, 1.042, 95%CI, 0.317-3.421; P>0.05) didn't achieve any statistical difference in OS in model 1. Moreover, marital status and treatment significantly correlated with all-cause mortality in model 2 (Table 3). Marital status divorced (vs married; HR, 2.672, 95%CI, 1.027-6.953; P=0.044) and treatment were significantly associated with increased all-cause mortality. Intriguingly, being divorced was associated with a higher risk of mortality than married status, while single (vs married; HR, 0.643, 95%CI, 0.327-1.264; P=0.200) was not statistical significant. And treatment chemotherapy+surgery+radiotherapy (vs others; HR, 0.096, 95%CI, 0.030-0.308; P<0.0001), surgery plus chemotherapy (vs others; HR, 0.081, 95%CI, 0.021-0.312; P<0.0001), radiotherapy plus chemotherapy (vs others; HR, 0.260, 95%CI, 0.115-0.587; P=0.001) were found to be protective interventions of survival in model 2. Generally, it seemed that divorced marital status, absence of positive treatment including any combination with surgery, chemotherapy, and radiotherapy had increased risk of mortality among OCBM patients.

**Figure 2:**
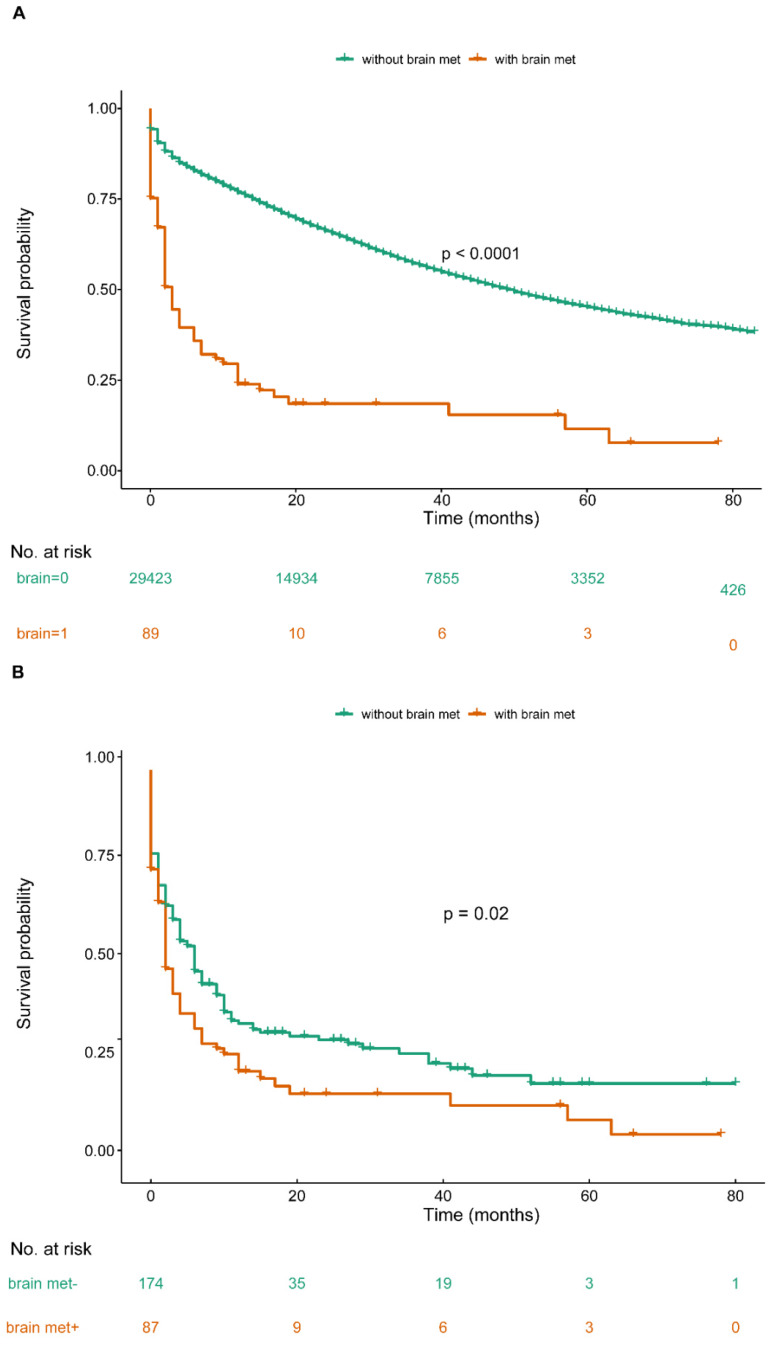
Overall survival among patients with or without de novo brain metastases at ovary cancer diagnosis before and after PSM (A. OS among OC patients with or without BM before PSM; B. OS among OC patients with or without BM after PSM).

**Figure 3:**
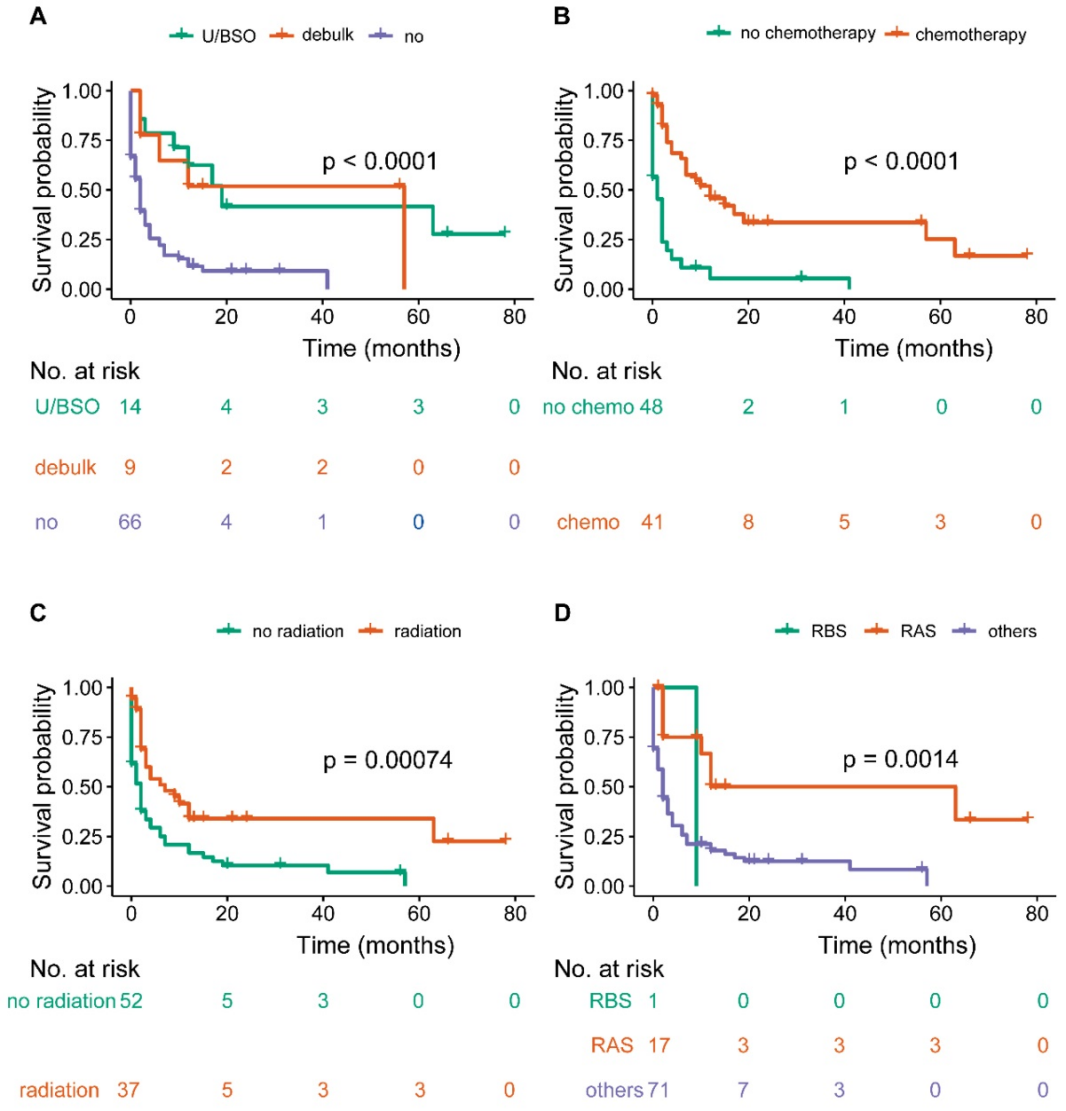
Overall survival among patients with OCBM at diagnosis (A, stratified by surgery, B, stratified by chemotherapy; C, stratified by radiation; D, stratified by sequence between surgery and radiation).

The median survival time of the OCBM cohort was 2.0 months (IQR: 0.0-10.0mo) (Table 1, Figure 2A). The median survival time for the patients with unilateral/bilateral (salpingo-) oophorectomy was 12.0 months (7.5-30.75mo), with cytoreductive surgery was 12.0 months (IQR: 2.0-35.5 mo), without surgery was 2.0 months (IQR: 0.0-4.0 mo) (Figure 3A). Median survival time of patients receiving chemotherapy was 9.0 months (IQR: 2.0-16.0 mo), while no chemotherapy was 1.0 month (IQR: 0.0-2.0mo) (Figure 3B). The median survival time for radiation-treated patients was 4.0 months (IQR: 2.0-12.0mo), among those without radiation was 1.5 months (IQR: 0.0-6.0 mo) (Figure 3C).

**Figure 4:**
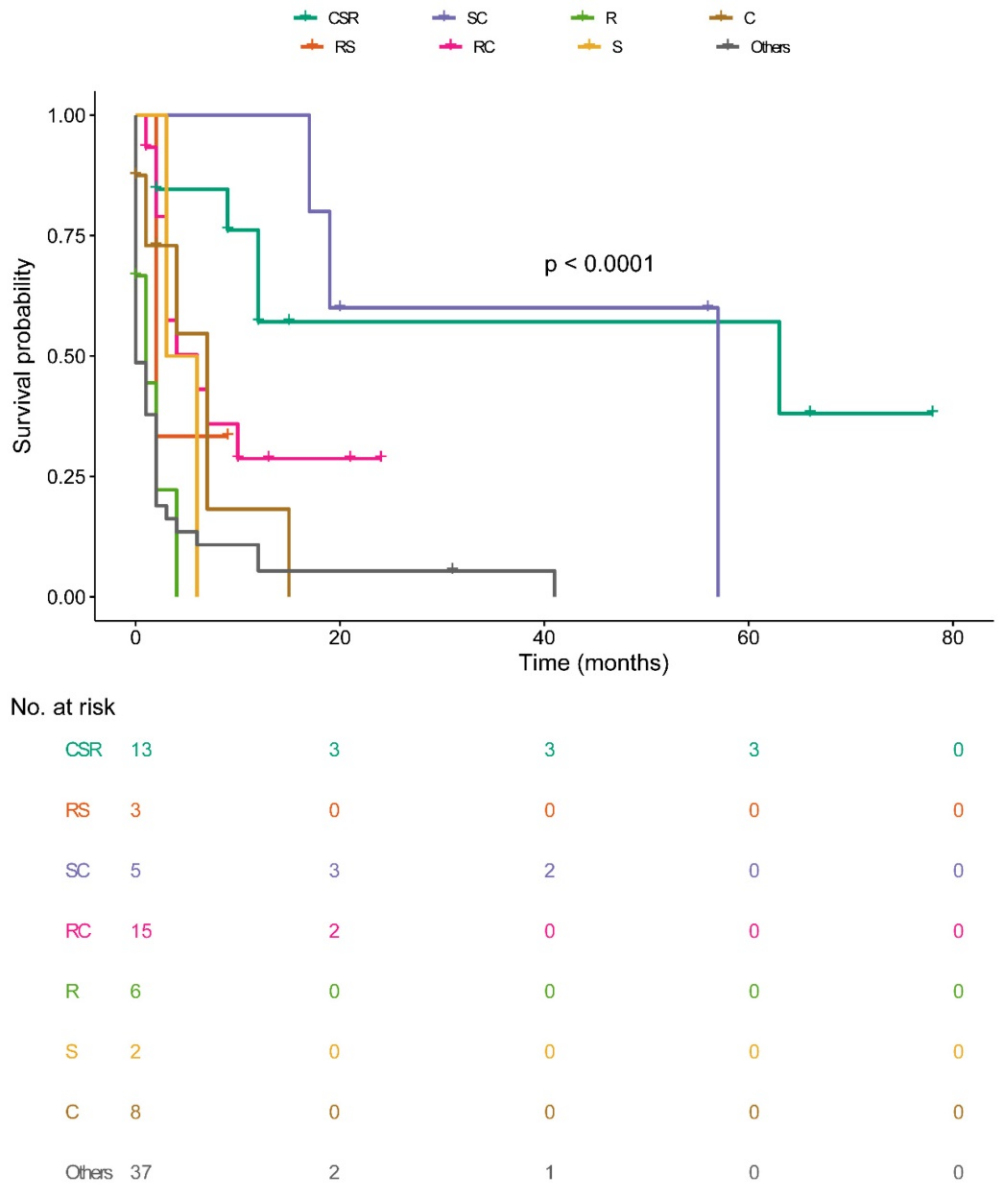
Overall survival among patients with OCBM at diagnosis stratified by treatment.

Median survival time among the sequence of radiotherapy and surgery indicated those received radiotherapies before surgery were 9.0 months (NA mo), radiation after surgery was 10.0 months (IQR: 2.0-14.0 mo), others were 2.0 months (IQR: 0.0-6.0 mo) (Figure 3D). Median survival time of CSR-treated patients was 12.0 months (IQR: 5.5-39.0mo), RS-treated was 2.0 months (IQR: 2.0-NA mo), SC-treated was 20.0 months (IQR:18.0-56.5mo), RC-treated was 4.0 months (IQR: 2.0-10.0mo), R-treated was 0.5 months (IQR: 0.0-2.5mo), S-treated was 4.5 months (IQR: 3.0-NAmo), C-treated was 3.0 months (IQR: 0.25-7.0mo), others was 0.0 month (IQR:0.0-2.0mo) (Figure 4). Survival analysis among the number of extracranial metastases was displayed in Figure S1 as supplementary.

### Survival analysis among PSM-matched cohort

Given the confounding factors between groups of with or without brain metastases, a 1:1 ratio PSM was used to balance the baseline clinical characteristics. There was no variable that achieved significant difference between case and controls (Table 4) after matching. In the matched cohort, we included 178 OC patients: 89 cases of brain met group and another 89 cases of without brain met group. Based upon the comparable variables between two groups, we conducted univariate Cox regression to draw a more accurate conclusion: ovary cancer with brain metastases contributed to poor prognosis for OC patients (Figure 1B, P<0.05). The median survival time of with or without brain metastases group was 2.0 months (IQR: 0.0-9.0 mo) and 5.0 months (IQR: 1.0-14.3 mo), respectively. 1:1 PSM based multivariate Cox regression identified 2 new factors (brain met and liver met), apart from the above prognostic factors for OS (Table 5).

We found that the presence of brain metastases at newly diagnosed ovarian cancer associated with shorter survival time compared with the propensity-score-matched control group in our study (Figure 1B). Also, the result indicated that patients receiving aggressive therapy benefited more in survival. The prognosis of patients treated with SC was best, while patients received other treatments had a poorer prognosis.

## Discussion

This study described the incidence and survival of the ovarian carcinoma patients with brain metastases at intial diagnosis based on the SEER database. The present study was the first time investigating both predictors and prognostic factors of de novo OCBM. Noteworthily, this population-based study also firstly employed the PSM analysis method to evaluate the role of brain metastases in ovarian carcinoma. BM was considered to be a rare and late event in ovarian carcinoma 3, it usually followed with high mortality, poor prognosis, causing colossal health burden and expensive medical costs 17. Although several systematic reviews and articles had been published upon the topic of ovarian cancer brain metastases 1, 3, 14, 18, sufficient evidence had not been found to provide clear guidelines on proper treatment, let alone the de novo brain metastases of ovarian carcinoma. In this circumstance, early detection and comprehensive treatment were of great significance, for it may alter the natural progression and improve overall survival. Thus, it is necessary to study OCBM patients in a large-scale cohort.

We found that 0.30% of patients with brain metastases at initial diagnosis of ovary cancer, and 12.02% was with metastatic disease at diagnosis, which were similar to previous study^19^, and a little lower than some researches 6, 7, 10, 17, 20-22. This may be because they counted not only patients with brain metastases at the time of diagnosis of ovarian cancer, but also patients with subsequent brain metastases. We identified predictors of OCBM using multivariate logistic regression. This study found that the unspecified histology, absence of surgery, no chemotherapy, and more extracranial metastasis sites increased the risk of developing OCBM in the entire cohort, which was similar to previous studies 8, 14, 18, 20, 23-25. However, Cohen et al. 10 held the opposite view that there was no association between the BM and the presence of other extracranial metastases.

Moreover, we also found that patients with bilateral laterality were more likely to be OCBM, which had never been reported before as we knew. Intriguingly, radiotherapy was found to be a higher risk of metastatic diseases to any site, including brain metastases, which might due to the fact that OCBM patients are usually in advanced disease when receiving radiation therapy as palliative treatment. However, only surgery and chemotherapy treatment had a lower risk to be OCBM, and our study did not show that positive or negative of CA125 elevation associated with OCBM, which were consistent to the research published before^18^, ^26^, however, was different with Divine et al. 25. These researches 1, 8, 27 showed that elder age, residence type and ethnicity were the risk factors for brain metastases, but in our study, these variables only achieved significance on the univariate logistic analysis.

This study indicated that bilateral laterality, unspecified histology, absence of surgery or chemotherapy, more extracranial metastasis might suffer a higher likelihood to be brain metastases among OC patients. Thus, these high-risk people might require a further examination at the initial diagnosis. Besides, patients without insurance should be encouraged to get screening regularly.

Furthermore, we found that divorced marriage, absence of surgery, absence of chemotherapy, and no insurance harmed overall survival among OCBM upon multivariate logistic regression analysis. And histology, radiotherapy and insurance status were not associated with prognosis which were similar to the previous study 28-30. We supposed that divorced patients went through more sufferings during the failed marriage, getting less treatment due to lacking their spouses' support, leading to poor survival. However, the unmarried status like single and widowed did not achieve statistical significance in overall survival in this study, which differed from the previous research. The extracranial metastases sites which showed decreased survivals had been reported 10, 15, 31, while in our study, extracranial metastases showed poorer survival among the entire cohort, but not in the OCBM subset. However, the reason remains unknown which needs further exploration.

To obtain satisfactory cytoreductive surgery and sensitive chemotherapy are the common treatment strategy for ovarian cancer 32, 33, which also applies to OCBM patients in this study. Surgery, radiation, and chemotherapy are the major treatment methods to prolong the patients' survival. Mostly, surgery supplemented with radiation therapy is a common choice. In our study, chemotherapy was the most common treatment in 89 patients (46.1%), which is slightly different from the previous research, for these had radiotherapy as the mostly used treatment 24. However, it is still controversial on the effect of chemotherapy for many chemotherapeutic drugs that cannot permeate the blood-brain barrier (BBB), which drastically limits the effectiveness. And some concerns that the use of systemic chemotherapy may corrupt the BBB. Thus, radiotherapy remains top choice for OC treatment, except for the brain metastases, which in turn helps to explain the former difference. There are two types of radiotherapy, which are commonly used, including stereotactic radiosurgery (SRS) and whole-brain radiotherapy (WBRT). Some authors 24, 34 reported that WBRT could cause physical and cognitive decline. A systemic retrospective study 35 confirmed that SRS had a better effect on the treatment of OCBM, with more adequately measured lesions, less-lethal of healthy cells as well as less adverse responses, compared to the WBRT. Besides, recent studies have shown that systemic chemotherapy for brain metastases showed favorable responses and prolonging their survival 6, 17, 18.

In model 1, the median survival time of OCBM patients decreased 2.5 months from chemotherapy to no chemotherapy (P<0.001). Moreover, median survival time decreased 10 months from U/BSO surgery to no surgery (P<0.001) and the prognosis for U/BSO surgery looks similar to the cytoreductive surgery (P>0.05). The median survival time from no radiotherapy to radiotherapy increased by 2.5 months (P<0.001). Besides, compared with surgery only (4.5 mo) (P>0.05) or chemotherapy only (3.0 mo) (P=0.031), patients who acquired surgery plus chemotherapy seemed to have longer median survival (20 mo). In model 2, median survival time of OCBM patients increased 12 months from others to RSC, 2 months from others to RS, decreased 20 months from SC to others, 3 months from C to others (P>0.05), 4.5 months from S to others (P>0.05), and there was little difference from others to RS or R. This result indicated that patients receiving multimodal therapy had significant benefits on survival. According to the results above, OCBM patients benefit more from chemotherapy and surgery, especially a combination of two, but radiotherapy achieved no statistical significance in overall survival analysis. The median survival of SC was the best, while patients with “others” treatment had the worst prognosis. The present study showed SC had the best prognosis, followed by CSR and RC among the comprehensive treatments for OCBM, but RS showed no statistical difference for overall survival, which might be meaningful for clinical practice. What's more, we found similar results in our PSM-matched analysis: in model 1, divorced marital status, absence of treatment including surgery and chemotherapy, liver met, and lung met were associated with more reduced overall survival among OCBM patients. Likewise, in model 2, single, divorced marital status showed poor prognosis, and combined treatment including CSR, SC, and RT was significantly associated with improved OS. Surgery or chemotherapy could also considerably reduce the risk of OCBM death. However, radiotherapy did not show statistical significance in improving the overall survival among OCBM patients based on the PSM-matched analysis.

### Limitations

Although this study is the large-scale multi-center study, but it has some limitations. Firstly, we only know the information of four metastatic sites, including the liver, bone, lung, and brain. This database has not included information on other metastatic sites, such as peritoneal metastases. Besides, we only knew the information of synchronous metastasis to the brain, consisting of a small part regarding those who might develop metachronous metastasis afterward. Secondly, some well-established covariates of survival such as comorbidities or performance status or even some tumor-related data such as stage, the types and numbers of brain metastases, the size of tumor, mammography screening, and treatment for comorbidities are not available in the database. Thirdly, the SEER database has not recorded the morbidity and mortality after treatment. Lastly, residence type was labeled at a county level, rather than a patient level, which may affect the results of the analysis.

This study firstly conducted a population-based analysis of OCBM patients as well as PSM-based analysis, to our knowledge. It provided valuable advice for patients with higher risk of OCBM to consider using MRI assessment. Besides, we also analyzed the prognostic factors of OCBM in the research. Additionally, the study compared the significance of different therapies for OCBM patients and provided recommendations for their clinical treatment. Moreover, this large-scale study used an efficient statistical method to emphasize the reliability of the impact of de novo brain metastases on survival in patients with OC.

## Supplementary Material

Supplementary figures and tables.

## Figures and Tables

**Table 1:** Clinical Characteristics of Patients for Ovarian Cancer Patients Diagnosed with and without Brain Metastases

Variables	Patients, No.			Proportion of Brain Metastases, %		Survival among Patients with Brain Metastases, Median (IQR), mo
	OC (N=29512)	OC with Brain Metastases (N=89)	OC without Brain Metastases (N=29423)	Among Entire cohort	Among Metastatic subset	
**Age (years)**						
Median (range)	62 (18-102)			-	--	
18-58	12359	28	12331	0.23	2.72	3(2-11.25)
≥59	17153	61	17092	0.36	2.42	2 (0-10)
**Marital status**						
Married	14188	35	14153	0.25	2.26	3(1-12)
Single	6565	22	6542	0.34	2.77	2(0-10.5)
Divorced	2990	8	2982	0.27	2.18	1 (0-2)
Widowed	4424	17	4408	0.38	2.51	2(0-7)
Unknown	1345	7	1338	0.52	4.38	4(2-10)
**Race**						
White	23792	70	23722	0.29	2.50	2(0-9)
Black	2692	12	2692	0.45	2.64	2(0.25-36)
Others	2852	5	2847	0.18	1.75	2(0-18.5)
Unknown	176	2	174	1.14	22.22	10.5(NA)
**Origin recode**						
Hispanic	4136	11	4125	0.27	2.43	6(2-12)
None-hispanic	25376	78	25298	0.31	2.52	2(0-10)
**Laterality**						
Unilateral	15441	32	15409	0.42	1.51	
Bilateral	9362	23	9339	0.25	1.24	6(2-15)
Unknown	4709	34	4675	0.72	2.05	1.5(0-6.25)
**Tumor staging**						
I	6966	0	6966	0	0	NA-
II	2435	0	2435	0	0	NA
III	10799	1	10798	0.01	33.33	6(NA)
IV	8239	87	8152	1.06	2.47	2(0-10)
Unknown	1073	1	1072	0.09	4.76	2(NA)
**T staging**						
T1	7811	11	7800	0.14	6.55	2(0-12)
T2	3724	9	3265	0.24	3.01	4(1-12.5)
T3	15084	26	15058	0.17	1.22	5(2-12.75)
Unknown	2893	43	2850	1.49	4.57	2(0-6)
**N staging**						
N0	20246	45	20201	0.22	2.73	2(0-12.5)
N1	6202	21	6181	0.34	1.91	2(0.5-9)
Unknown	3064	23	3041	0.75	2.89	3(1-7)
**Histology**						
Epithelial neoplasms	26303	67	26236	0.25	2.12	2(1-10)
Gonadal neoplasms	1297	2	1295	0.15	5.00	21.5(12-NA)
Others	1071	3	1068	0.28	2.07	12(2-NA)
Unknown	841	17	824	2.02	8.37	0(0-2.5)
**Treatment^a^**						
Others	3371	37	3334	1.10	3.98	0(0-2)
CSR	213	13	200	6.10	29.55	12(5.5-39)
RS	24	3	21	12.50	60.00	2(2-NA)
SC	17127	5	17122	0.03	0.35	20(18-56.5)
RC	77	15	62	19.48	31.25	4(2-10)
R	38	6	32	15.79	30.00	0.5(0-2.5)
S	5647	2	5645	0.04	1.18	4.5(3-NA)
C	3015	8	3007	0.27	0.90	3(0.25-7)
**Surgery**						
U/BSO	12146	14	12132	0.12	2.77	12(7.5-30.75)
Cytoreductive	10360	9	10351	0.09	0.83	12(2-35.5)
Others	505	0	505	0	0	NA
No	6501	66	6435	1.02	3.50	2(0-4)
**Radiotherapy**						
No	29160	52	29108	0.18	1.52	1.5 (0-6)
Yes	352	37	315	10.51	31.62	4(2-12)
**Chemotherapy**						
No	9080	48	9032	0.53	4.27	1(0-2)
Yes	20432	41	20391	0.20	1.69	9(2-16)
**Radiation Sequence with surgery^b^**						
RBS	15	1	14	6.67	14.29	9(NA)
RAS	235	17	218	7.23	35.42	10(2-14)
RBAS	1	0	1	0	0	NA
Others	29261	71	29190	0.24	2.03	2(0-6)
**Bone Met**						
No	29160	61	29099	0.21	1.90	2(0-11)
Yes	310	24	286	7.74	7.74	3(0-8.5)
Unknown	42	4	38	9.52	18.18	2.5(0.5-9.75)
**Liver Met**						
No	27249	62	27187	0.23	4.47	2(0-10.5)
Yes	2120	21	2099	0.99	0.99	2(0.5-5)
Unknown	143	6	137	4.20	15.00	2(0-12.75)
**Lung Met**						
No	27538	49	27489	0.18	2.85	2(0.5-11)
Yes	1773	37	1736	2.09	2.09	2(0-8)
Unknown	201	3	198	1.49	5.36	0(NA)
**CA125**						
Normal	2654	4	2650	0.15	5.13	16.5(6-54.75)
Elevated	20241	49	20192	0.24	1.80	2(0-8)
Unknown	6617	36	6581	0.54	4.85	2(0-11.5)
**Insurance situation**						
No	1096	7	1089	0.64	4.55	2(0-2)
Yes	27927	77	27846	0.28	2.31	2(0-12)
Unknown	489	5	484	1.02	8.33	3(2.5-6.5)
**Residence type**						
Rural	482	1	481	0.21	1.67	2(NA)
Urban	3376	12	3364	0.36	3.10	1(0-2.75)
Metropolitan	25630	76	25554	0.30	2.46	2.5(0.25-12)
Unknown	24	0	24	0	0	NA

Abbreviations: OC: ovary cancer; IQR: interquartile range; CI: confidence interval. ^a^ including CSR: chemotherapy, surgery, and radiotherapy, RS: radiotherapy and surgery, SC: surgery and chemotherapy, RC: radiotherapy and chemotherapy, R: radiotherapy alone, S: surgery alone, C: chemotherapy alone, others: other treatment except for radiotherapy, surgery, and chemotherapy. ^b^including RBS: radiation before surgery, RAS: radiotherapy after surgery, RBAS: radiotherapy before and after surgery, others: without surgery or radiotherapy or unknown sequence.

**Table 2:** Multivariate Logistic Regression for the Patients with Brain Metastases at Diagnosis of Ovary Cancer

Variables	Patients, No			Among Entire Cohort		Among Subset with Metastatic Disease	
	Patients (N=29512)	With Metastatic Disease (N=3546)	With Brain Metastases (N=89)	OR (95%CI)	*P* value	OR (95%CI)	*P* value
**Age (years)**							
18-58	12359	1028	28	Reference		Reference	
≥59	17153	2518	61	1.331(0.760-2.329)	0.317	0.742(0.412-1.337)	0.321
**Laterality**							
Unilateral	15441	1192	32	Reference		Reference	
Bilateral	9362	1130	23	2.251(1.206 -4.204)	0.011	1.649(0.831-3.270)	0.152
Unknown	4709	1224	34	0.742(0.403-1.366)	0.338	0.564(0.298-1.067)	0.078
**T staging**							
T1	7811	168	11	Reference		Reference	
T2	3724	299	9	0.607(0.225-1.636)	0.324	0.466(0.151-1.444)	0.186
T3	15084	2138	26	0.706(0.301-1.657)	0.424	0.455(0.179-1.152)	0.097
Unknown	2893	942	43	1.555(0.645-3.750)	0.325	0.826(0.335-2.037)	0.679
**N staging**							
N0	20246	1650	45	Reference		Reference	
N1	6202	1101	21	0.689(0.371-1.279)	0.238	0.837(0.452-1.551)	0.571
Unknown	3064	795	23	0.678(0.364-1.262)	0.220	0.682(0.356-1.306)	0.248
**Histology**							
Epithelial neoplasms	26303	3158	67	Reference		Reference	
Gonadal neoplasms	1297	40	2	0.744(0.156-3.540)	0.710	2.052(0.333-12.655)	0.439
Others	1071	145	3	0.762(0.201-2.886)	0.689	1.280(0.345-4.754)	0.712
Unknown	841	203	17	2.406(1.226-4.725)	0.016	2.568(1.246-5.293)	0.011
**Surgery**							
U/BSO	12146	506	14	Reference		Reference	
Cytoreductive	10360	1078	9	0.648(0.253-1.659)	0.368	0.500(0.175-1.428)	0.195
Others	505	76	0	0.000(0.000-NA)	0.993	0.000(0.000-NA)	0.997
No	6501	1886	66	4.047(1.702 -9.624)	0.002	1.766(0.708-4.404)	0.222
**Radiotherapy**							
No	29160	117	52	Reference		Reference	
Yes	352	3429	37	32.268 (15.423-67.508)	<0.0001	27.036(12.397-58.963)	<0.0001
**Chemotherapy**							
No	9080	2421	48	Reference		Reference	
Yes	20432	1125	41	0.503(0.287-0.881)	0.016	0.432(0.236-0.791)	0.006
**Radiation Sequence with surgery**							
RBS	15	7	1	Reference		Reference	
RAS	235	48	17	2.496(0.259-24.077)	0.429	1.576(0.141-17.601)	0.712
RBAS	1	1	0	0.000(0.000-NA)	1.000	0.000(0.000-NA)	1.000
Others	29261	3490	71	0.564(0.057-5.593)	0.625	0.994(0.089-11.057)	0.996
**Bone Met**							
No	29160	310	61	Reference		Reference	
Yes	310	3214	24	5.095(2.737-9.488)	<0.0001	0.706(0.350-1.424)	0.331
Unknown	42	22	4	7.271(1.811-29.190)	0.005	9.748(2.073-45.845)	0.004
**Liver Met**							
No	27249	2120	62	Reference		Reference	
Yes	2120	1386	21	1.072(0.581-1.977)	0.824	0.144(0.073-0.281)	<0.0001
Unknown	143	40	6	5.266(1.706-16.250)	0.004	3.836(1.104-13.323)	0.034
**Lung Met**							
No	27538	1773	49	Reference		Reference	
Yes	1773	1717	37	5.328(3.124-9.085)	<0.0001	0.339(0.178-0.643)	0.001
Unknown	201	56	3	1.627(0.369-7.176)	0.521	0.700(0.110-4.464)	0.706
**CA125**							
Normal	2654	78	4	Reference		Reference	
Elevated	20241	2726	49	1.437(0.439-4.712)	0.549	0.957(0.251-3.647)	0.949
Unknown	6617	742	36	2.234(0.674-7.400)	0.188	1.507(0.390-5.823)	0.552
**Insurance situation**							
No	1096	3332	7	Reference		Reference	
Yes	27927	154	77	0.506(0.201-1.274)	0.148	0.674(0.250-1.816)	0.436
Unknown	489	60	5	1.751(0.473-6.484)	0.402	1.721(0.379-7.817)	0.482

Abbreviations: OR: odds ratio; CI: confidence interval.

**Table 3:** Multivariate Cox Regression for the Ovary Cancer Patients with Brain Metastases

Variables	Patients, No.		All-Cause Mortality (model 1)		All-Cause Mortality (model 2)	
	Patients (N=29512)	With Brain Metastases (N=89)	HR (95%CI)	P value	HR (95%CI)	P value
**Marital status**						
Married	14188	35	Reference		Reference	
Single	6565	22	0.679(0.351-1.313)	0.249	0.643(0.327-1.264)	0.200
Divorced	2990	8	2.688(1.102-6.560)	0.030	2.672(1.027-6.953)	0.044
Widowed	4424	17	0.710(0.355-1.419)	0.332	0.659 (0.324-1.341)	0.250
Unknown	1345	7	0.515(0.185-1.429)	0.202	0.485(0.170-1.384)	0.176
**Histology**						
Epithelial neoplasms	26303	67	Reference		Reference	
Gonadal neoplasms	1297	2	0.215(0.028-1.670)	0.142	0.215(0.028-1.675)	0.142
Others	1071	3	1.183(0.144-9.737)	0.876	0.998(0.105-9.486)	0.999
Unknown	841	17	0.921(0.480-1.766)	0.804	0.906(0.465-1.767)	0.906
**Treatment**						
Others	3371	37	NA	NA	Reference	NA
CSR	213	13	NA	NA	0.096(0.030-0.308)	<0.0001
RS	24	3	NA	NA	0.283(0.060-1.342)	0.112
SC	17127	5	NA	NA	0.081 (0.021-0.312)	<0.0001
RC	77	15	NA	NA	0.260(0.115-0.587)	0.001
R	38	6	NA	NA	1.015(0.357-2.886)	0.978
S	5647	2	NA	NA	0.207 (0.040-1.086)	0.063
C	3015	8	NA	NA	0.428(0.167-1.101)	0.078
**Surgery**						
U/BSO	12146	14	Reference		NA	NA
Cytoreductive	10360	9	1.042(0.317-3.421)	0.946	NA	NA
Others	505	0	NA	NA	NA	NA
No	6501	66	3.712(1.519-9.075)	0.004	NA	NA
**Radiotherapy**					NA	NA
No	29160	52	Reference		NA	NA
Yes	352	37	0.893(0.481 -1.657)	0.720	NA	NA
**Chemotherapy**						
No	9080	48	Reference		NA	NA
Yes	20432	41	0.341(0.169-0.687)	0.003	NA	NA
**Insurance situation**						
No	1096	7	Reference		Reference	
Yes	27927	77	0.776(0.315-1.912)	0.582	0.754(0.281-2.020)	0.574
Unknown	489	5	0.965(0.255-3.655)	0.958	0.984 (0.242-4.006)	0.984

Abbreviations: CI: confidence interval.

**Table 4:** Baseline Characteristics of Patients for Ovarian Cancer Patients Diagnosed with and without Brain Metastases before and after PSM

Variables	Before matching			After matching		
	OC without Brain Metastases (N=29423, %)	OC with Brain Metastases (N=89, %)	*P* value	OC without Brain Metastases (N=89, %)	OC with Brain Metastases (N=89, %)	*P* value
**Age (years)**			0.072			0.884
Median (SD)	61.26 (15.20)	64.16 (13.81)		63.81 (17.83)	64.16 (13.81)	
**Marital status**			0.295			0.554
Married	14153 (48.1)	35 (39.3)		35 (39.3)	35 (39.3)	
Single	6542 (22.2)	22 (24.7)		24 (27.0)	22 (24.7)	
Divorced	2982 (10.1)	8 (9.0)		5 (5.6)	8 (9.0)	
Widowed	4408 (15.0)	17 (19.1)		22 (24.7)	17 (19.1)	
Unknown	1338 (4.5)	7 (7.9)		3 (3.4)	7 (7.9)	
**Race**			0.058			0.826
White	23722 (80.6)	70 (78.7)		73 (82.0)	70 (78.7)	
Black	2680 (9.1)	12 (13.5)		12 (13.5)	12 (13.5)	
Others	2847 (9.7)	5 (5.6)		3 (3.4)	5 (5.6)	
Unknown	174 (0.6)	2 (2.2)		1 (1.1)	2 (2.2)	
**Origin recode**			0.766			0.826
None-hispanic	25298 (86.0)	78 (87.6)		76 (85.4)	78 (87.6)	
**Laterality**			<0.001			0.405
Unilateral	15409 (52.4)	32 (36.0)		30 (33.7)	32 (36.0)	
Bilateral	9339 (31.7)	23 (25.8)		17 (19.1)	23 (25.8)	
Unknown	4675 (15.9)	34 (38.2)		42 (47.2)	34 (38.2)	
**T staging**			<0.001			0.592
T1	7800 (26.5)	11 (12.4)		7 (7.9)	11 (12.4)	
T2	3715 (12.6)	9 (10.1)		11 (12.4)	9 (10.1)	
T3	15058 (51.2)	26 (29.2)		32 (36.0)	26 (29.2)	
Unknown	2850 (9.7)	43 (48.3)		39 (43.8)	43 (48.3)	
**N staging**			<0.001			0.849
N0	20201 (68.7)	45 (50.6)		48 (53.9)	45 (50.6)	
N1	6181 (21.0)	21 (23.6)		18 (20.2)	21 (23.6)	
Unknown	3041 (10.3)	23 (25.8)		23 (25.8)	23 (25.8)	
**Histology**			<0.001			0.849
Epithelial neoplasms	26236 (89.2)	67 (75.3)		60 (67.4)	67 (75.3)	
Gonadal neoplasms	1295 (4.4)	2 (2.2)		4 (4.5)	2 (2.2)	
Others	1068 (3.6)	3 (3.4)		10 (11.2)	3 (3.4)	
Unknown	824 (2.8)	17 (19.1)		15 (16.9)	17 (19.1)	
**Surgery**			<0.001			0.176
U/BSO	12132 (41.2)	14 (15.7)		10 (11.2)	14 (15.7)	
Cytoreductive	10351 (35.2)	9 (10.1)		13 (14.6)	9 (10.1)	
Others	505 (1.7)	0 (0.0)		2 (2.2)	0 (0.0)	
No	6435 (21.9)	66 (74.2)		64 (71.9)	66 (74.2)	
**Radiotherapy**						1.000
	315 (1.1)	37 (41.6)	<0.001	37 (41.6)	37 (41.6)	
**Chemotherapy**						0.653
	20391 (69.3)	41 (46.1)	<0.001	45 (50.6)	41 (46.1)	
**Radiation Sequence with surgery**			<0.001			0.559
RBS	14 (0.0)	1 (1.1)		1 (1.1)	1 (1.1)	
RAS	218 (0.7)	17 (19.1)		23 (25.8)	17 (19.1)	
RBAS	1 (0.0)	0		0	0	
Others	29190 (99.2)	71 (79.8)		65 (73.0)	71 (79.8)	
**Treatment**			<0.001			0.469
CSR	200 (0.7)	13 (14.6)		18 (20.2)	13 (14.6)	
RS	21 (0.1)	3 (3.4)		2 (2.2)	3 (3.4)	
SC	17122 (58.2)	5 (5.6)		1 (1.1)	5 (5.6)	
RC	62 (0.2)	15 (16.9)		12 (13.5)	15 (16.9)	
R	32 (0.1)	6 (6.7)		5 (5.6)	6 (6.7)	
S	5645 (19.2)	2 (2.2)		4 (4.5)	2 (2.2)	
C	3007 (10.2)	8 (9.0)		14 (15.7)	8 (9.0)	
Others	3334 (11.3)	37 (41.6)		33 (37.1)	37 (41.6)	
**Bone Met**			<0.001			0.186
No	29099 (98.9)	61 (68.5)		70 (78.7)	61 (68.5)	
Yes	286 (1.0)	24 (27.0)		14 (15.7)	24 (27.0)	
Unknown	38 (0.1)	4 (4.5)		5 (5.6)	4 (4.5)	
**Liver Met**			<0.001			0.781
No	27187 (92.4)	62 (69.7)		65 (73.0)	62 (69.7)	
Yes	2099 (7.1)	21 (23.6)		20 (22.5)	21 (23.6)	
Unknown	137 (0.5)	6 (6.7)		4 (4.5)	6 (6.7)	
**Lung Met**			<0.001			0.298
No	27489 (93.4)	49 (55.1)		52 (58.4)	49 (55.1)	
Yes	1736 (5.9)	37 (41.6)		30 (33.7)	37 (41.6)	
Unknown	198 (0.7)	3 (3.4)		7 (7.9)	3 (3.4)	
**CA125**			<0.001			0.127
Normal	2650 (9.0)	4 (4.5)		0 (0.0)	4 (4.5)	
Elevated	20192 (68.6)	49 (55.1)		50 (56.2)	49 (55.1)	
Unknown	6581 (22.4)	36 (40.4)		39 (43.8)	36 (40.4)	
**Insurance situation**			0.001			0.167
No	1089 (3.7)	7 (7.9)		2 (2.2)	7 (7.9)	
Yes	27850 (94.7)	77 (86.5)		84 (94.4)	77 (86.5)	
Unknown	484 (1.6)	5 (5.6)		3 (3.4)	5 (5.6)	
**Residence type**			0.905			0.819
Rural	481 (1.6)	1 (1.1)		2 (2.2)	1 (1.1)	
Urban	3364 (11.4)	12 (13.5)		13 (14.6)	12 (13.5)	
Metropolitan	25554 (86.9)	76 (85.4)		74 (83.1)	76 (85.4)	
Unknown	24 (0.1)	0 (0.0)		0 (0.0)	0 (0.0)	

Abbreviations: PSM: Propensity Score Matching; SD: Standard Deviation

**Table 5:** Univariate and multivariate Cox Regression for the Brain Metastases of Ovary Cancer after PSM.

Variables	Univariate			Multivariate in model 1			Multivariate in model 2			
	HR (95%CI)	P value		HR (95%CI)	P value			HR (95%CI)	P value	
**Age (years)**										
18-58	Reference			Reference			Reference			
≥59	1.575(1.086-2.283)	0.017		0.719 (0.418-1.237)		0.233	0.752(0.435-1.298)			0.306
**Marital status**										
Married	Reference			Reference			Reference			
Single	0.762(0.470-1.233)		0.268	0.599(0.344-1.043)		0.070	0.548(0.317-0.948)			0.031
Divorced	2.163(1.201-3.894)		0.010	1.977(0.977-4.003)		0.058	1.882(0.890-3.981)			0.098
Widowed	1.665(1.081-2.563)		0.021	1.073(0.629-1.829)		0.797	1.002(0.583-1.723)			0.994
Unknown	0.976(0.476-1.999)		0.946	0.567(0.238-1.347)		0.199	0.532(0.220-1.288)			0.162
**Race**										
White	Reference			NA		NA	NA			NA
Black	0.829(0.513-1.340)	0.443		NA		NA	NA			NA
Others	1.470(0.766-2.820)	0.247		NA		NA	NA			NA
Unknown	0.466(0.065-3.348)	0.448		NA		NA	NA			NA
**Origin recode**										
Hispanic	Reference			Reference			Reference			NA
Non-hispanic	1.535(0.805-2.928)	0.193		0.798(0.369-1.727)		0.298	0.923(0.428-1.990)			0.837
**Laterality**										
Unilateral	Reference			Reference			Reference			NA
Bilateral	0.777(0.472-1.278)	0.320		0.694(0.379-1.270)		0.236	0.743(0.401-1.378)			0.346
Unknown	1.153(0.788-1.687)	0.463		1.119(0.679-1.846)		0.659	1.112(0.673-1.837)			0.678
**Tumor staging**										
I	Reference			NA		NA	NA			NA
II	0.904(0.000-2.159E67)	0.999		NA		NA	NA			NA
III	1856.939(0.000-2.6481E61)	0.912		NA		NA	NA			NA
IV	3401.073(0.000-4.837E+61)	0.905		NA		NA	NA			NA
Unknown	1933.002(0.000-2.751E+61)	0.912		NA		NA	NA			NA
**T staging**										
T1	Reference			Reference		NA	Reference			NA
T2	0.759(0.355-1.621)	0.477		0.794(0.335-1.882)		0.600	0.872(0.365-2.082)			0.757
T3	0.890(0.476-1.662)	0.714		0.921(0.430-1.973)		0.832	1.007(0.481-2.108)			0.985
Unknown	1.047(0.576-1.902)	0.880		0.687(0.315-1.499)		0.346	0.697(0.324-1.503)			0.358
**N staging**										
N0	Reference			Reference		NA	Reference			NA
N1	1.203(0.779-1.856)	0.405		1.165(0.690-1.965)		0.568	1.183(0.697-2.011)			0.533
Unknown	1.473(0.975-2.225)	0.066		1.013(0.609-1.685)		0.960	1.006(0.601-1.684)			0.981
**Histology**										
Epithelial neoplasms	Reference			Reference			Reference			NA
Gonadal neoplasms	0.324(0.045-2.323)	0.262		0.192(0.024-1.508)		0.117	0.176(0.022-1.392)			0.100
Others	0.696(0.323-1.499)	0.355		0.793(0.302-2.079)		0.637	0.976(0.376-2.535)			0.960
Unknown	1.293(0.824-2.030)	0.264		0.592(0.336-1.043)		0.070	0.606(0.342-1.072)			0.085
**Treatment**										
Others	Reference			NA		NA	Reference			
CSR	0.209(0.112-0.390)	<0.0001		NA		NA	0.104(0.045-0.236)			<0.0001
RS	0.555(0.136-2.275)	0.414		NA		NA	0.350 (0.069-1.783)			0.206
SC	0.224(0.081-0.620)	0.004		NA		NA	0.061(0.018-0.212)			<0.0001
RC	0.357(0.215-0.595)	<0.0001		NA		NA	0.231(0.124-0.429)			<0.0001
R	0.863(0.443-1.683)	0.666		NA		NA	0.710(0.307-1.641)			0.423
S	0.340(0.136-0.847)	0.021		NA		NA	0.231(0.070-0.763)			0.016
C	0.502(0.285-0.884)	0.017		NA		NA	0.310(0.158-0.608)			0.001
**Surgery**										
U/BSO	Reference			Reference			NA			NA
Cytoreductive	1.117(0.483-2.582)	0.797		1.120 (0.427-2.938)		0.818	NA			NA
Others	0.000(0.000- 4.8509E168)	0.959		0.000(0.000-7.797E+183)		0.961	NA			NA
No	2.743(1.564-4.810)	<0.0001		3.141(1.433-6.886)		0.004	NA			NA
**Radiotherapy**										
No	Reference			Reference			NA			NA
Yes	0.499(0.347-0.717)	<0.0001		0.817(0.462-1.442)		0.485	NA			NA
**Chemotherapy**										
No	Reference			Reference			NA			NA
Yes	0.3720.260-0.531()	<0.0001		0.298(0.180-0.495)		<0.0001	NA			NA
**Radiation Sequence with surgery**										
Others	Reference			Reference			NA			NA
RBS	0.365(0.090-1.481)	0.158		1.283(0.247-6.660)		0.767	NA			NA
RAS	NA	NA		NA		NA	NA			NA
RBAS	0.417(0.250-0.697)	0.001		1.151(0.536-2.473)		0.718	NA			NA
**Brain Met**										
No	Reference			Reference			Reference			
Yes	1.327(0.943-1.866)	0.104		1.600(1.042-2.458)		0.032	1.653(1.076-2.541)			0.022
**Bone Met**										
No	Reference			Reference			Reference			
Yes	1.186(0.780-1.805)	0.425		0.951 (0.554 -1.630)		0.854	1.040(0.596-1.814)			0.891
Unknown	1.877(0.999-3.528)	0.050		0.977(0.307-3.112)		0.969	0.974(0.297-3.193)			0.965
**Liver Met**										
No	Reference			Reference			Reference			
Yes	1.756(1.187-2.598)	0.005		1.899(1.119-3.223)		0.018	1.941(1.140-3.303)			0.015
Unknown	1.995(0.999-3.987)	0.050		1.506(0.535-4.236)		0.438	1.584(0.556-4.508)			0.389
**Lung Met**										
No	Reference			Reference			Reference			
Yes	1.834(1.274-2.641)	0.001		1.374(0.826-2.284)		0.221	1.305(0.785-2.169)			0.305
Unknown	1.949(0.934-4.068)	0.075		1.500(0.455-4.941)		0.505	1.436(0.438-4.712)			0.550
**CA125**										
Normal	Reference			Reference		NA	Reference			NA
Elevated	2.338(0.850-6.428)	0.100		1.543(0.502-4.747)		0.449	1.502(0.488-4.621)			0.478
Unknown	2.063(0.747-5.702)	0.163		1.398(0.457-4.275)		1.398	1.387(0.452-4.259)			0.567
**Insurance situation**										
No	Reference			Reference			Reference			
Yes	0.491(0.274-0.878)	0.017		0.701 (0.323-1.522)		0.369	0.704(0.324-1.531)			0.376
Unknown	0.644(0.210-1.980)	0.443		0.897(0.234-3.446)		0.875	0.938(0.244-3.607)			0.926
**Residence type**										
Rural	Reference			Reference		NA	Reference			NA
Urban	0.599(0.177-2.023)	0.409		0.956(0.202-4.525)		0.955	0.892(0.182-4.373)			0.888
Metropolitan	0.428(0.135-1.359)	0.150		0.587(0.129-2.677)		0.491	0.535(0.112-2.547)			0.432
Unknown	NA	NA		NA		NA	NA			NA
										
