# Supplementary material for: Prognostic Factors among Brain Metastases in Newly Diagnosed Ovary Cancer: A Large Real-world Study
Source: J Cancer. 2020 May 18;11(15):4625–40. doi: 10.7150/jca.44494 (PMC7255353; doi:10.7150/jca.44494)

Table S1. Univariate Logistic Regression for the Ovary Cancer Patients with Brain Metastases among the Entire Cohort.

Variables	among the Entire Cohort.		among the Metastatic Subset.	
	OR (95%CI)	*P* value	OR (95%CI)	P value
**Age (years)**				
18-58	Reference		Reference	
⩾59	1.572 (1.004-2.460)	0.048	0.887(0.563-1.395)	0.603
**Marital status**				
Married	Reference		Reference	
Single	1.360(0.797-2.319)	0.260	1.233(0.718-2.116)	0.448
Divorced	1.085(0.503-2.341)	0.836	0.964(0.443-2.096)	0.926
Widowed	1.560(0.873-2.787)	0.133	1.116(0.621-2.006)	0.714
Unknown	2.116(0.938-4.772)	0.071	1.979(0.864-4.531)	0.106
**Race**				
White	Reference		Reference	
Black	1.517(0.821-2.803)	0.183	1.058(0.569-1.967)	0.859
Others	0.595(0.240-1.476)	0.263	0.693(0.277-1.732)	0.433
Unknown	3.895(0.948-16.010)	0.059	11.131(2.271-54.542)	0.003
**Origin recode**				
Hispanic	Reference		Reference	
None-hisnic	1.156(0.614-2.176)	0.653	1.037(0.547-1.965)	0.912
**Laterality**				
Unilateral	Reference		Reference	
Bilateral	1.186 (0.694-2.028)	0.533	0.753(0.438-1.295)	0.305
Unknown	3.502(2.159-5.681)	<0.0001	1.036(0.635-1.690)	0.888
**Tumor staging**				
I	Reference	NA	-	-
II	2.907(2.600-3.249)	<0.0001	-	-
III	6.207(5.718-6.737)	<0.0001	Reference	NA
IV	12.758(11.756-13.846)	<0.0001	0.051(0.005-0.564)	0.015
Unknown	11.390(10.181-12.742)	<0.0001	0.100(0.004-2.287)	0.149
**T staging**				
T1	Reference		Reference	
T2	1.718(0.711-4.149)	0.229	0.443(0.180-1.092)	0.077
T3	1.224(0.605-2.479)	0.574	0.176(0.085-0.362)	<0.0001
Unknown	10.699(5.510-20.775)	<0.0001	0.683(0.345-1.354)	0.275
**N staging**				
N0	Reference		Reference	
N1	1.525(0.908-2.562)	0.111	0.694(0.411-1.171)	0.171
Unknown	3.395(2.051-5.619)	<0.0001	1.063(0.638-1.769)	0.815
**Histology**				
Epithelial neoplasms	Reference		Reference	
Gonadal neoplasms	0.605(0.148-2.471)	0.484	2.428(0.574-10.273)	0.228
Others	1.100(0.345-3.503)	0.872	0.975(0.303-3.137)	0.966
Unknown	8.079(4.723-13.818)	<0.0001	4.217(2.427-7.326)	<0.0001

**Treatment**				
Others	Reference		Reference	
RS	12.873(3.679-45.036)	<0.0001	36.203(5.871-223.250)	<0.0001
SC	0.026(0.010-0.067）	<0.0001	0.084(0.033-0.215)	<0.0001
RC	21.800(11.376-41.777)	<0.0001	10.971(5.485-21.944)	<0.0001
R	16.895(6.665-42.830)	<0.0001	10.344(3.762-28.437)	<0.0001
S	0.032(0.008-0.133)	<0.0001	0.287(0.069-1.204)	0.088
C	0.240(0.111-0.516)	<0.0001	0.219(0.102-0.474)	<0.0001
CSR	5.857(3.064-11.195)	<0.0001	10.121(4.895-20.925)	<0.0001
**Surgery**				
U/BSO	Reference		Reference	
Cytoreductive	0.753(0.326-1,741)	0.508	0.296(0.127-0.688)	0.005
Others	0.000(0.000-NA)	0.994	0.000(0.000-NA)	0.997
NO	8.888(4.989-15.835)	<0.0001	1.274(0.710-2.288)	0.417
**Radiotherapy**				
No	Reference		Reference	
Yes	65.751（42.519-101.676）	<0.0001	30.036(18.655-48.360)	<0.0001
**Chemotherapy**				
No	Reference		Reference	
Yes	0.378（0.249-0.574）	<0.0001	0.387(0.253-0.590)	<0.0001
**Radiation Sequence with surgery**				
RBS	Reference		Reference	
RAS	1.092(0.135-8.808)	0.934	3.290(0.365-29.638)	0.288
RBAS	0.000(0.000-NA)	1.000	0.000(0.000-NA)	1.000
Others	0.034(0.004-0.262)	0.001	0.125(0.015-1.048)	0.055
**Bone Met**				
No	Reference		Reference	
Yes	40.031(24.612-65.109)	<0.0001	4.337（2.664-7.063）	<0.0001
Unknown	50.214(17.389-145.002)	<0.0001	11.486(3.775-34.946)	<0.0001
**Liver Met**				
No	Reference		Reference	
Yes	4.387(2.669-7.210)	<0.0001	0.214(0.120-0.352)	<0.0001
Unknown	19.204(8.170-45.140)	<0.0001	3.769(1.525-9.311)	0.004
**Lung Met**				
No	Refence		Reference	
Yes	11.957(7.781-18.374)	<0.0001	0.726(0.471-1.118)	0.146
Unknown	8.500(2.627-27.498)	<0.0001	1.927(0.582-6.380)	0.283
**Extracranial Metastatic sites to bone, lung, and liver, No.**				
0	Reference		Reference	
1	5.694(3.345-9.691)	<0.0001	3.174(1.852-5.438)	<0.0001
2	21.506(11.861-38.996)	<0.0001	19.806(10.439-37.579)	<0.0001
3	92.269(41.227-206.502)	<0.0001	1.240E+11(0.000-NA)	0.999
Unknown	15.497(6.846-35.082)	<0.0001	5.372(2.334-12.368)	<0.0001

**CA125**				
Normal	Reference		Reference	
Elevated	1.608(0.580-4.458)	0.362	0.339(0.119-0.963)	0.042
Unknown	3.624(1.289-10.192)	0.015	0.943(0.327-2.724)	0.914
**Insurance situation**				
No	Reference		Reference	
Yes	0.430(0.198-0.935)	0.033	0.497(0.225-1.096)	0.083
Unknown	1.607(0.508-5.089)	0.420	1.909(0.582-6.267)	0.286
**Residence type**				
Rural	Reference		Reference	
Urban	1.716(0.223-13.225)	0.604	1.888(0.241-14.789)	0.545
Metropolitan	1.431(0.199-10.308)	0.722	1.486(0.203-10.868)	0.696
Unknown	NA	0.999	NA	NA

Table S2. Univariate Cox Regression for the Ovary Cancer Patients among the Entire Cohort.

Variables	Patients, No.		All-Cause Mortality among Entire Cohort		All-Cause Mortality among Brain Metastases	
	Patients (N=29512)	With Brain Metastases （N=89）	HR (95%CI)	P value	HR (95%CI)	P value
**Age (years)**						
18-58	12359	28	Reference		Reference	
≥59	17153	61	2.564(2.462-2.670)	<0.0001	1.227(0.739-2.036)	0.430
**Marital status**						
Married	14188	35	Reference		Reference	
Single	6565	22	1.114(1.061-1.170)	<0.0001	1.019(0.538-1.929)	0.954
Divorced	2990	8	1.392(1.310-1.478)	<0.0001	2.860(1.258-6.504)	0.012
Widowed	4424	17	2.537(2.423-2.658)	<0.0001	1.404(0.749-2.632)	0.290
Unknown	1345	7	1.259(1.152-1.376)	<0.0001	0.846(0.322-2.223)	0.734
**Race**						
White	23792	70	Reference		Reference	
Black	2692	12	1.348(1.273-1.428)	<0.0001	0.774(0.386-1.549)	0.468
Others	2852	5	0.735(0.686-0.787)	<0.0001	1.061(0.384-2.931)	0.910
Unknown	176	2	0.299(0.195-0.459)	<0.0001	0.397(0.055-2.881)	0.361
**Origin recode**						
Hispanic	4136	11	Reference		Reference	
Non-hispanic	25376	78	1.215(1.149-1.284)	<0.0001	1.518(0.693-3.325)	0.297
**Laterality**						
Unilateral	15441	32	Reference		Reference	
Bilateral	9362	23	1.519(1.456-1.585)	<0.0001	0.799(0.426-1.500)	0.485
Unknown	4709	34	4.846(4.634-5.069)	<0.0001	1.360(0.787-2.349)	0.270
**Tumor staging**						
I	6966	0	Reference		Reference	
II	2435	0	2.907(2.600-3.249)	<0.0001	NA	NA
III	10799	1	6.207(5.718-6.737)	<0.0001	NA	NA
IV	8239	87	12.758(11.756-13.846)	<0.0001	NA	NA
Unknown	1073	1	11.390(10.181-12.742)	<0.0001	NA	NA
**T staging**						
T1	7811	11	Reference		Reference	
T2	3724	9	3.175(2.922-3.450)	<0.0001	1.024(0.363-2.891)	0.964
T3	15084	26	5.403(5.057-5.774)	<0.0001	0.953(0.410-2.215)	0.911
Unknown	2893	43	12.754(11.814-13.768)	<0.0001	1.353(0.615-2.977)	0.452
**N staging**						
N0	20246	45	Reference		Reference	
N1	6202	21	1.554(1.489-1.622)	<0.0001	1.219(0.667-2.227)	0.519
Unknown	3064	23	3.258(3.099-3.424)	<0.0001	1.369(0.778-2.407)	0.276
**Histology**						
Epithelial neoplasms	26303	67	Reference		Reference	
Gonadal neoplasms	1297	2	0.180(0.150-0.216)	<0.0001	0.373(0.051-2.716)	0.330
Others	1071	3	1.836(1.695-1.990)	<0.0001	0.340(0.047-2.467)	0.286

Unknow	841	17	3.785(3.494-4.099)	<0.0001	2.422(1.374-4.272)	0.002
**Treatment**						
CSR	213	13	Reference		Reference	
RS	24	3	1.345(0.742-2.440)	0.329	3.782(0.703-20.352)	0.121
SC	17127	5	0.532(0.441-0.642)	<0.0001	1.163(0.275-4.913)	0.837
RC	77	15	2.335(1.665-3.276)	<0.0001	2.963(0.962-9.121)	0.058
R	38	6	3.297(2.179-4.989)	<0.0001	9.509(2.563-35.281)	0.001
S	5647	2	0.402(0.331-0.487)	<0.0001	5.195(0.961-28.089)	0.056
C	3015	8	2.523(2.087-3.051)	<0.0001	4.350(1.262-14.986)	0.020
Others	3371	37	5.715(4.730-6.906)	<0.0001	8.415(3.097-22.865)	<0.0001
**Surgery**						
U/BSO	12146	14	Reference		Reference	
Cytoreductive	10360	9	2.332(2.218-2.451)	<0.0001	1.297(0.410-4.100)	0.658
Others	505	0	2.399(2.088-2.756)	<0.0001	NA	NA
No	6501	66	11.462(10.908-12.046)	<0.0001	4.302(1.881-9.841)	0.001
**Radiotherapy**						
No	352	37	Reference		Reference	
Yes	29160	52	1.605(1.396-1.846)	<0.0001	0.455(0.273-0.759)	0.003
**Chemotherapy**						
No	20432	41	Reference		Reference	
Yes	9080	48	0.625(0.602-0.649)	<0.0001	0.266(0.157-0.450)	<0.0001
**Radiation Sequence with surgery**						
RBS	15	1	Reference		Reference	
RAS	235	17	0.694(0.364-1.322)	0.267	0.379(0.046-3.107)	0.366
RBAS	1	0	4.291(0.549-33.522)	0.165	NA	NA
Others	29261	71	0.535(0.288-0.995)	0.048	1.353(0.186-9.824)	0.765
**Brain Met**						
No.	89	89	Reference		NA	NA
Yes	29413	0	4.059(3.208-5.135)	<0.0001	NA	NA
**Bone Met**						
No	310	24	Reference		Reference	
Yes	29160	61	3.859(3.395-4.388)	<0.0001	1.025(0.599-1.755)	0.928
Unknown	42	4	3.807(2.732-5.306)	<0.0001	1.429(0.513-3.984)	0.495
**Liver Met**						
No	2120	21	Reference		Reference	
Yes	27249	62	2.762(2.615-2.918)	<0.0001	1.481(0.861-2.546)	0.156
Unknown	143	6	3.157(2.614-3.813)	<0.0001	1.567(0.665-3.694)	0.304
**Lung Met**						
No	1773	37	Reference		Reference	
Yes	27538	49	2.967(2.799-3.145)	<0.0001	1.368(0.840-2.230)	0.208
Unknown	201	3	4.142(3.554-4.827)	<0.0001	1.971(0.601-6.461)	0.263
**CA125**						
Normal	2654	4	Reference		Reference	
Elevated	20241	49	3.552(3.220-3.918)	<0.0001	3.448(0.820-14.493)	0.091

Unknown	6617	36	3.083(2.781-3.418)	<0.0001	3.085(0.732-13.003)	0.125
**Insurance situation**						
No	1096	7	Reference		Reference	
Yes	27927	77	0.986(0.898-1.082)	0.759	0.422(0.188-0.948)	0.037
Unknown	489	5	1.221(1.036-1.440)	0.017	0.464(0.135-1.599)	0.224
**Residence type**						
Rural	482	1	Reference		Reference	
Urban	3376	12	0.999(0.867-1.150)	0.986	1.140(0.146-8.876)	0.900
Metropolitan	25630	76	0.858(0.751-0.980)	0.024	0.526(0.072-3.844)	0.527
Unknown	24	0	0.988(0.553-1.767)	0.968	NA	NA

Figure S1. Overall survival among patients with OCBM at diagnosis stratified by the number of metastatic diseases to any distant site.

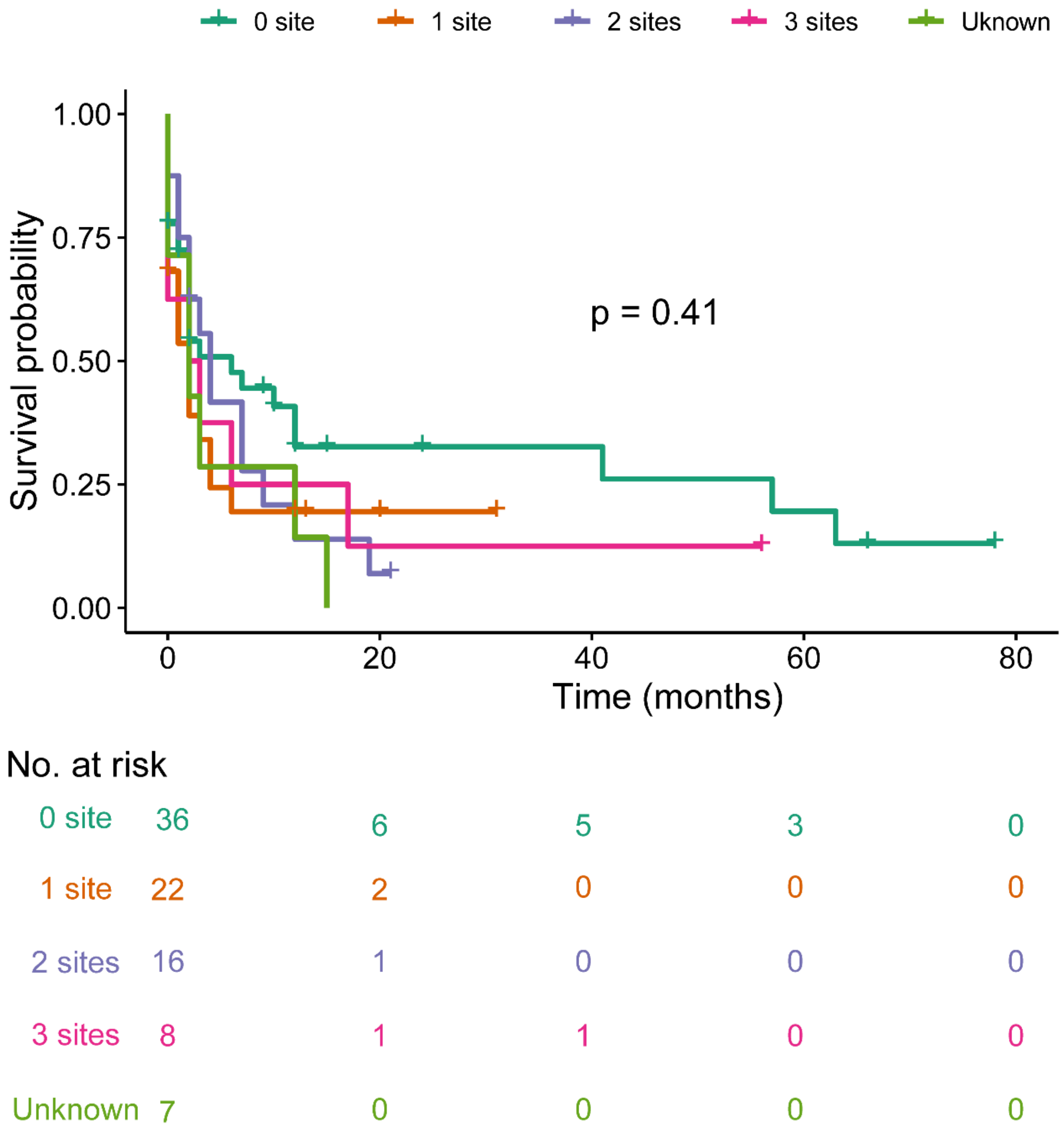

Supplement: Supplementary file 1 — Supplementary figures and tables. [file jcav11p4625s1.pdf]
